# The effectiveness of an online‐based psychosocial program for parents of children with neurodevelopmental disorders – a randomized controlled trial

**DOI:** 10.1111/camh.70044

**Published:** 2025-11-05

**Authors:** Ana Pardo‐Salamanca, Soledad Gómez, Cristina Santamarina, Gemma Pastor, Carmen Berenguer

**Affiliations:** ^1^ Universitat de Valencia Valencia Spain; ^2^ Universidad Catolica de Valencia San Vicente Martir Campus de Valencia San Juan Bautista Valencia Spain; ^3^ Universidad Politécnica de Valencia Valencia Spain

**Keywords:** Online, psychosocial intervention, ADHD, ASD, RCT

## Abstract

**Background:**

Neurodevelopmental disorders like autism and ADHD may disrupt family and child functioning. Although well‐established psychosocial treatment programs exist, access to these interventions remains limited for many families. This randomized controlled trial (RCT) primarily evaluates the effectiveness of an online psychosocial program (INPSYD) in improving parental domains (stress, coping, and social support). The secondary aim is to assess its effects on children's domains, including behavior, executive functioning, sleep, and family dynamics.

**Methods:**

This study employed a two‐arm, mixed‐methods RCT design involving families of children diagnosed with autism or ADHD (mean age 9.28 ± 1.6; 81.1% males), (clinicaltrials.gov, NCT06303791). Participants were randomized to either the intervention group or the active control group (a structured, non‐therapeutic intervention). Outcomes were collected at baseline (before randomization), at 12 weeks post‐randomization, and at 6 months follow‐up after randomization.

**Results:**

A total of 82 participants were randomized in February 2024 (42 to the intervention group and 40 to the active control group). At post‐intervention, participants in the intervention group scored statistically significantly better than those in the control group on measures of parenting stress (*p* < .01, $\eta_{p}^{2}$ = .29), coping skills (*p* < .01, $\eta_{p}^{2}$ = .24), and perceived social support (*p* = .025, $\eta_{p}^{2}$ = .10). Between‐group comparisons also revealed statistically significant differences favoring the intervention group in child social functioning, executive functioning, and family dynamics. No statistically significant between‐group differences were found for sleep problems or learning behavior. These between‐group effects were maintained at the follow‐up assessment.

**Conclusion:**

The results of this study indicate that the INPSYD parenting program is effective for improving parenting stress, coping skills and children's executive functioning and social‐behavioral outcomes in our sample.

**Key Practitioner Message**

**What is currently known?**

- Neurodevelopmental disorders like autism and ADHD disrupt family and child functioning. Psychosocial treatments often face limitations.

**What has been shown?**

- This RCT found that the online INPSYD program reduced parenting stress, improved coping skills, and enhanced social support for parents. Children showed better social skills, executive functioning, and family dynamics, with sustained benefits 3 months after post‐intervention.

**What is the significance of this for clinical practice?**

- Clinicians and policymakers should consider integrating such digital interventions into routine care for families of children with neurodevelopmental disorders, particularly in settings where access to in‐person services is limited.

## Introduction

Attention‐deficit/hyperactivity disorder (ADHD) and autism spectrum disorder (ASD) are among the most prevalent neurodevelopmental disorders in school‐aged children, exerting significant and long‐lasting impacts on affected individuals and their families due to their severity and chronic nature. The diagnostic prevalence of ADHD is estimated to range between 8% and 11% in children and adolescents aged 5–17 years in the United States (Danielson et al., 2018) and reaches 9.92% in Spain within the same age group (Bosch et al., 2022). Conversely, the diagnostic prevalence of ASD has quadrupled over the past decade, with a rate of 1 in 68 children in the United States (US Department of Health and Human Services, 2016). These high prevalence rates underscore the considerable healthcare and educational costs associated with these conditions (Chorozoglou et al., 2015). While ADHD and ASD differ in core symptomatology – ADHD being defined by challenges in attention, hyperactivity, and impulsivity, and ASD by deficits in communication, social interaction, and repetitive behaviors with restricted interests – both disorders share underlying similarities. Neuroscientific research points to genetic predispositions and alterations in brain circuits that affect executive functions, somatic motor functions, dorsal attention, and visual processing, impacting critical processes such as planning, inhibition, decision‐making, and working memory (Proal, González‐Olvera, Blancas, Chalita, & Castellanos, 2013).

Both ASD and ADHD negatively affect various domains of functioning, complicating long‐term individual adaptation. In the social and adaptive domains, ADHD is associated with deficits in peer and family interactions, leading to peer rejection due to challenges in sharing, initiating, cooperating, or waiting for turns. These social deficits contribute to long‐term emotional difficulties, increased risk behaviors, and impairments in overall functioning (Mrug et al., 2012).

On the other hand, autistic children experience social challenges rooted in limited emotional reciprocity and reduced interest in social interactions (APA, 2013).

Moreover, both disorders are linked to deficits in executive functioning (Barkley, 2011; Pennington & Ozonoff, 1996) and impairments in social cognition processes, such as theory of mind and emotion recognition (Bora & Pantelis, 2016).

Psychosocial interventions based on social learning theory and cognitive‐behavioral techniques (CBT) have proven effective in managing symptoms associated with ADHD and ASD, often complementing pharmacological treatments, which tend to show limited efficacy in addressing associated difficulties such as parenting stress or behavioral challenges (Daley et al., 2014). Notably, recent work has emphasized the need to prioritize psychosocial approaches – particularly parent‐mediated programs integrating psychoeducation and CBT – as a first‐line treatment for school‐aged children with neurodevelopmental conditions (Chacko, Merrill, Kofler, & Fabiano, 2024; Young et al., 2020).

Parent training interventions delivered in person have received substantial empirical support from randomized controlled trials (RCTs). However, systematic reviews point to several limitations in this body of literature, including heterogeneity in outcome measures, small and inconsistent sample sizes, and limited long‐term follow‐up data (Cheng, Smith, Butler, Taylor, & Clayton, 2023; Conrad et al., 2021; Marquet‐Doleac, Biotteau, & Chaix, 2024). The effectiveness of these programs is influenced not only by child characteristics (e.g. externalizing behaviors, attention difficulties) but also by parental stress levels and systemic challenges, such as inadequate institutional support (McStay, Dissanayake, Scheeren, Koot, & Begeer, 2014). Furthermore, a growing body of research advocates moving beyond a purely medical model toward a bio‐psycho‐social framework that recognizes the role of social and contextual factors in shaping outcomes for neurodiverse children and their families (Hsiao, 2018; Sulla et al., 2023). Despite the availability of evidence‐based interventions, many families face significant barriers to access, including geographic distance, scheduling conflicts, and limited availability of services (Danielson et al., 2018; Tan‐MacNeill, Smith, Johnson, Chorney, & Corkum, 2021). These limitations have fueled increasing interest in telehealth and online parent‐mediated programs.

Recent meta‐analyses and reviews demonstrate that digital interventions can be effective, acceptable, and feasible for families of children diagnosed with ASD or ADHD. For instance, Pan, Kuo, and Kuo (2023) reported that telehealth‐delivered programs improved children's social communication and adaptive behavior. Similarly, Tan‐MacNeill et al. (2021) found that internet‐based interventions were well‐received by families and led to meaningful developmental gains. Florean, Dobrean, Păsărelu, Georgescu, and Milea (2020) confirmed the efficacy of online parenting programs in reducing behavioral problems, and Gonzalez et al. (2023) showed that group‐based telehealth training for ADHD was comparable to in‐person delivery, with added accessibility benefits.

These findings support the integration of digital delivery formats in parent training programs, particularly as a means to address logistical and structural barriers. However, more high‐quality trials are needed to assess their efficacy in diverse populations and over time.

The current study responds to this gap by evaluating a fully online, synchronous psychosocial program specifically designed for families of children diagnosed with ASD and ADHD. While established in‐person programs such as The Incredible Years (Webster‐Stratton, Dababnah, & Olson, 2018) have demonstrated effectiveness in early childhood behavioral outcomes, this trial extends the scope by targeting both parental well‐being and broader child functioning using a multicomponent, digital format.

The primary aim of this randomized controlled trial (RCT) is to assess the efficacy of the online program – delivered via live video sessions – in improving parental outcomes, specifically stress, coping strategies, and social support. The secondary aim is to explore its impact on child outcomes, including social skills, executive functioning, sleep quality, family relationships, and learning behaviors.

Outcomes are evaluated immediately after the intervention (T1: post‐intervention) and at follow‐up (T2), conducted 6 months after randomization, which corresponds to 3 months post‐intervention.

To our knowledge, this is the first study to examine the impact of a fully online, synchronous, multicomponent psychosocial program targeting both parental and child domains in families of autistic children and children diagnosed with ADHD. By integrating psychoeducation, stress management, CBT tools, and social communication training, this study contributes to the development of more comprehensive and accessible interventions for neurodiverse populations.

## Methods

### Design

This study was a two‐arm, single‐blinded, parallel RCT design involving families of children diagnosed with autism or ADHD.

The trial was registered at clinical trials.gov with the identifier NCT06303791 (https://clinicaltrials.gov/study/NCT06303791).

### Participants

The present study enrolled 90 families of boys and girls with ASD without intellectual disabilities and ADHD over a 1.5‐year period (2021–2023), with an age range between 7 and 11 years (47 autistic children without intellectual disabilities, and 43 children with ADHD). The selected age range reflects the developmental period during which children with autism or ADHD typically begin to face increasing demands in executive functioning, social interaction, and behavioral regulation. This range also aligns with prior research on parent‐mediated interventions (Marquet‐Doleac et al., 2024). Families and children were recruited through specialized psychoeducational centers in Spain, which distributed information about the study both on‐site and via their official websites. This approach enabled interested families to learn about the study and choose to participate voluntarily.

The inclusion criteria were as follows: (1) to have a clinical diagnosis of either autism spectrum disorder (ASD) or attention‐deficit/hyperactivity disorder (ADHD). Diagnosis of ASD according to the Autism Diagnostic Interview – Revised (ADI‐R; Rutter, Le Couteur, & Lord, 2006), and/or the Autism Diagnostic Observation Schedule, Generic (ADOS G; Lord, Rutter, DiLavore, & Risi, 2000) confirmed by a child psychologist or ADHD of any presentation according to the Diagnostic and Statistical Manual of Mental Disorders, 5th Edition (DSM‐5; APA, 2013) for parents and teachers; (2) IQ > 80 according to the K‐BIT (Kaufman & Kaufman, 2000); (3) age of children between 7 and 11 years, either sex; (4) informed consent of the parents and the children available; (5) parents' age greater than or equal to 18 years; (6) responsibility and legal capacity in parents and internet access. Exclusion criteria included children with any medical or psychiatric condition, an IQ of less than 80, motor or sensory deficits, and children whose families had received any psychosocial treatment before. Eight families were excluded after applying the eligibility criteria.

### Intervention

The intervention program and evaluation were conducted by expert psychologists specializing in neurodevelopmental disorders. The program's content was based on three main approaches, each of which had separately demonstrated evidence in the treatment of these neurodevelopmental disorders: psychoeducation and stress management, cognitive‐behavioral techniques, and social and communication skills techniques (Dahl et al., 2020; DuPaul, Chronis‐Tuscano, Danielson, & Visser, 2019; Tan‐MacNeill et al., 2021).

Parent intervention sessions were conducted in a synchronous virtual format to maximize program adherence and overcome logistical barriers related to physical distances, work commitments, and childcare. The intervention involved online sessions conducted exclusively with parents. Psychologists instructed them in specific behavioral techniques to be applied at home with their children. Sessions combined guidance and supervision, allowing psychologists to explain strategies, monitor parental understanding, and provide feedback to support effective implementation.

Specifically, the psychosocial intervention group consisted of five successive groups of 8–10 families, receiving 12 weekly sessions lasting 90 min each. Session integrity was ensured by creating a digital manual explicitly describing all procedures used in the intervention program.

The active control group participated in 12 weekly 90‐min online sessions designed to facilitate open discussion and peer support in a non‐directive format. Importantly, this was not treatment as usual, as no psychotherapy, psychoeducation, or specific psychosocial techniques were provided. The therapist's role was limited to guiding the conversation without delivering any therapeutic content.

The sessions were conducted by the same two individuals from the research team in both groups. Additionally, the integrity of the sessions was ensured through the use of a structured manual that clearly outlined all procedures and content to be delivered during the intervention. All sessions were recorded, and an independent team member reviewed session recordings and therapist adherence logs following a checklist to verify that each group received a consistent and equivalent set of information.

### Procedure

Before the first intervention session, parents were provided with a tutorial for the video conferencing platform (Zoom, www.zoom.us) to familiarize themselves with the software and minimize potential risks. Participants could access online meetings through a hyperlink or by entering the provided ID and password to join the session. Continuous contact via phone and virtual means was maintained with families to provide immediate assistance.

Each family attended three assessment sessions in person in a room of the faculty of psychology, of approximately 40–45 min each. Assessment took place at (T0) baseline/pre‐intervention (immediately before randomization), (T1) post‐intervention at 12 weeks after randomization, and (T2) follow‐up at 6 months after randomization.

### Outcomes

#### Primary outcomes – Parental domains

The primary outcomes were the effects of the intervention program on three parental domains as parenting stress, positive coping, and social support. The parenting stress was measured using the Parenting Stress Index *– Short Form* (PSI‐SF; Abidin, 1990; adapted to Spanish by Díaz‐Herrero, López‐Pina, Pérez‐López, de la Nuez, & Martínez‐Fuentes, 2011). The PSI‐SF is a self‐report questionnaire of 36 items that measures total parenting stress. The 36 items are distributed in three subscales of 12 items each (parental distress (e.g. ‘I feel trapped by my responsibilities as a parent’), parent–child dysfunctional interaction (e.g. ‘Sometimes I feel my child doesn't like me and doesn't want to be close to me’), and difficult child (e.g. ‘My child gets upset easily over the smallest thing’)) rated on a 5‐point Likert‐type response scale. The total score of PSI‐SF completed by parents was used in this study. The PSI‐SF presents good psychometric properties, and it is an effective measure for use with high‐risk families (Barroso, Hungerford, Garcia, Graziano, & Bagner, 2016). The internal consistency for the total scale was high in our sample (Cronbach's *α* = .92).

The positive coping was measured using the *Brief COPE* (Carver, Scheier, & Weintraub, 1989; Spanish adaptation, Morán, Landero & González, 2010), a 28‐item parent report instrument that measures the usage frequencies of maladaptive and adaptive coping strategies. Items are rated on a 4‐point rating scale ranging from *I haven't been doing this at all* to *I've been doing this a lot*. In this study, the Brief COPE was analyzed using one domain (Positive coping; e.g. ‘I've been trying to see it in a different light, to make it seem more positive’) as derived in Hastings et al. (2005). Positive coping included the Brief COPE items for the use of humor and positive reframing, and one item each from the acceptance and emotional social support scales. Internal consistency evaluations of the positive coping domain were adequate, with Cronbach's coefficient alpha of .83 (Morán, Landero, & González, 2010).

The social support was measured using the *Social Functional Support Questionnaire Duke‐UNC* (Broadhead, Gehlbach, Degruy, & Kaplan, 1988; Spanish adaptation; Bellón & Luna del Castillo, 1996). This questionnaire was filled out by parents and measures the perception of the availability of help from family and friends in difficult situations, as well as the ease of communication with them (e.g. ‘I receive invitations to go out and do things with other people’). The instrument comprises 11 questions with a Likert‐type scale ranging from 1 = *much less than I would like* to 5 = *as much as I would like*. Scores of less than 32 indicate low perceived social support. The present study used the direct total score. The internal consistency was good (Cronbach's *α* = .83) (Bellón & Luna del Castillo, 1996).

#### Secondary outcomes – Children domains

Secondary outcomes assessed included children's domains: (a) Social and behavioral difficulties as measured by the *Strengths and Difficulties Questionnaire* (SDQ; Goodman, 1997; Spanish adaptation by Rodríguez‐Hernández et al., 2012). The SDQ was filled out by parents to rate a wide range of psychopathological symptoms and prosocial behavior in children and adolescents between 4 and 16 years old. The questionnaire consists of five subscales (emotional symptoms, hyperactivity/inattention, conduct problems, peer problems and prosocial behavior). A total difficulties score (SDQ total; e.g. ‘Often has temper tantrums or hot tempers’, ‘Rather solitary, tends to play alone’) comprising the first four subscale scores indicates the overall extent of a child's psychopathological problems. Each of the five scales incorporates three response alternatives (not true, somewhat true, very true). Higher scores indicate more difficulties. The SDQ presents adequate psychometric properties with adequate reliability (between .73 and .76) (Goodman, 1997; Rodríguez‐Hernández et al., 2012); (b) Effect on executive functioning as measured by *The Behavioral Rating Inventory of Executive Function* (BRIEF‐2 Family Report; Gioia, Isquith, Guy, & Kenworthy, 2015). The BRIEF is a questionnaire used to assess daily executive functions. It consists of 63 items that are rated on a frequency scale, ranging from *never* (1) to *frequently* (3). For this study, the Global Executive Composite (GEC) representing overall executive functioning was utilized (e.g. ‘Does not realize that certain actions bother others’, ‘Has trouble getting started on homework or tasks’). Raw scores are converted into age‐corrected *T*‐scores, with a score greater than 70 on any of the scales indicating clinically significant difficulties. The BRIEF‐2 Family has demonstrated high internal consistency for the Index and Composite scores, with Cronbach's alpha ranging from .90 to .97 (Gioia et al., 2015); Effect on children's sleep problems, as measured by the Sleep Disturbance Scale for Children (SDSC; Bruni et al., 1996; Spanish adaptation by Pagerols et al., 2023). The SDSC is a 26‐item parent‐reported questionnaire designed to assess sleep disturbances in children aged 6–16 years. Each item is rated on a 5‐point scale, ranging from *never* to *always*, resulting in a continuous variable between 26 and 130. Higher scores indicate more severe sleep disturbance symptoms. The first two items measure total sleep time and latency to sleep onset, while the remaining items assess the frequency of sleep‐related behaviors across six subscales. The total sleep disturbances score is obtained by summing scores across all subscales (e.g. ‘After waking up in the night, the child has difficulty falling asleep again’). The scale has good internal consistency, with a reliability coefficient of *α* = .79 (Bruni et al., 1996); (d) Effect on family functioning as measured by *The Weiss Functional Impairment Rating Scale‐Parent Form* (WFIRS‐P) (Weiss, Brooks, Iverson, & Lee, 2007). The WFIRS‐P is a 50‐item scale that requires parents to rate the impact of their child's emotional or behavioral problems on six separate domains: Family, school and learning, life skills, child's self‐concept, social activities, and risky activities. Each item is rated on a 4‐point scale from 0 (*never or not at all*) to 3 (*very often or very much*) or rated as *not applicable*. The mean of all scored items for each domain was computed. The score of the family was used in this study (e.g. ‘Causing fighting in the family’). The scale has been psychometrically validated with an internal consistency Cronbach's *α* = .80 (Gajria et al., 2015) and effects on children's learning behavior as measured by *The Learning Behaviors Scale* (LBS; McDermott, Leigh, & Perry, 2002). The LBS is a teacher‐report questionnaire designed to measure student behaviors related to effective and efficient learning. The Learning Behaviors Scale contains 29 items, 6 items with positive wording and the remaining items with relatively negative wording in order to reduce response sets. Items are rated on a 3‐point Likert scale (0 = does not apply, 1 = sometimes applies, 2 = most often applies). High scores indicate good learning behavior, and low scores are interpreted as faulty learning behavior. Based on the manual, 25 of the 29 items were used to produce a total score and four subscales (e.g. ‘Adopts a don't‐care attitude to success or failure’). Total and subscale raw scores are converted to normalized *T* scores (*M* = 50, *SD* = 10). The internal consistency coefficient is high for the total score (*α* = .93) (Canivez & Beran, 2011; McDermott et al., 2002).

### Sample size

Sample size was calculated using a priori sample size calculator for one‐way ANOVA using G*power v. 3.1.9.4 setting the size of Type I error (significance level) at .05 (*α*) and the power of the study at 80%, anticipating an effect size (Cohen *d*) of .8 (Evans, Owens, Wymbs, & Ray, 2018; Tan‐MacNeill et al., 2021).

### Randomization

This randomized controlled trial studied the efficacy of an online‐based psychosocial intervention (INPSYD) compared with an active control group for 12 weeks, in parents of children diagnosed with ASD and ADHD. A single‐blind procedure was implemented in this study. Although the research team did not disclose group allocation to participants, it was not possible to fully blind them, as the differences between the structured therapeutic sessions and the non‐directive support groups were likely recognizable. However, the researchers responsible for data collection and entry were blinded to group assignments to minimize potential bias.

Families/children meeting the inclusion criteria were randomly assigned to the experimental and control groups. Block randomization was applied to each of the diagnostic groups (ASD, ADHD) to ensure that they were of approximately similar size (1:1 ratio) in the treatment and control conditions. After determining the size of each block in the sample (#ASD and #ADHD) the treatment allocation of each participant was done randomly. Participants in the intervention and control conditions were instructed to complete the intervention. Although recruitment occurred progressively, randomization was conducted in a single batch once the target sample size was achieved, with eligibility reconfirmed immediately prior to allocation. Finally, 82 families were randomly assigned (42 intervention group and 40 control group) using a computerized program for simple randomization.

### Data analysis

Statistical analyses were performed using the Statistical Package for the Social Sciences (SPSS) software, version 26.00 (SPSS Inc., Chicago, IL, USA). The distribution of the variables was analyzed, as well as their fit to the normal distribution curve, by applying Kolmogorov–Smirnov tests. Both the intervention group and the control group were assessed at baseline and post‐intervention. To assess for differences in parents' outcomes (stress level, positive coping and social support) and children's outcomes (SDQ, BRIEF, Sleep problems, WFIRS‐Family, and LBS) between intervention and control groups from pre‐ to post‐intervention as well as at follow‐up, two‐way mixed analysis of variances (ANOVAs) were conducted with group (intervention or control) as the between‐subjects factor and time (pre‐ or post‐intervention, follow‐up) as the within‐subjects factor. To control for Type I error associated with the analysis of multiple primary outcomes, the Holm–Bonferroni correction was applied. Partial eta squared ($\eta_{p}^{2}$) was used as a measure of effect size; values of less than .06 are considered small, between .06 and .14 are considered medium, and above .14 are considered large. Effect sizes of change were also calculated within each group from baseline/pre‐intervention to post‐intervention and follow‐up periods using Cohen's *d*. As Cohen (1988) suggested, *d* = 0.2 is considered a small effect size, 0.5 a medium effect size, and 0.8 a large effect size. There were no missing data in this study; all participants completed the assessments at baseline/pre‐intervention, post‐intervention, and follow‐up, with no item‐level omissions or attrition across time points.

## Results

**Figure 1:**
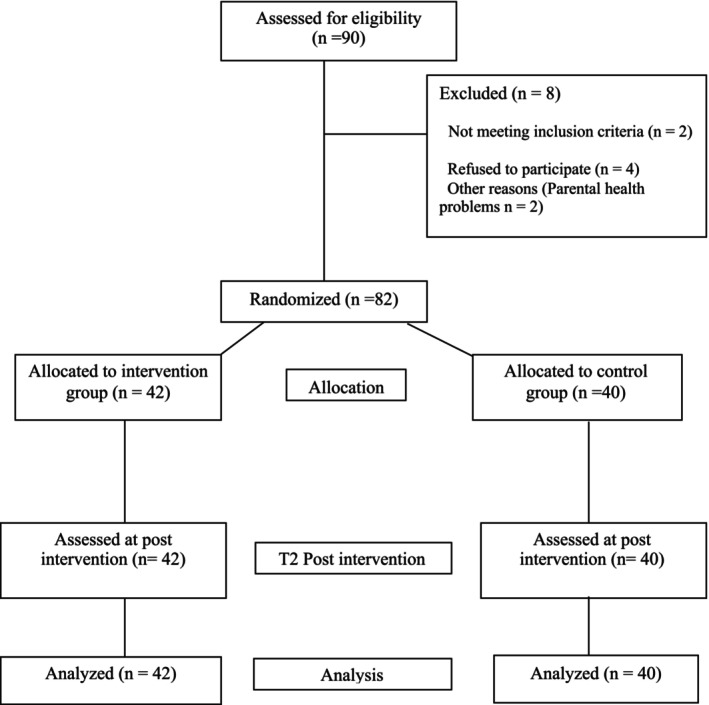
CONSORT diagram to illustrate study recruitment, random assignment, and data analysis

The participant inclusion process is shown in the flowchart (Figure 1) following the CONSORT checklist (Montgomery et al., 2018). A total of 82 participants were randomized in February 2024 (42 to the intervention group, 40 to the active control group). Table 1 provides the sample's sociodemographic baseline characteristics.

**Table 1:** Demographic characteristics

	Intervention group (*n* = 42)	Control group (*n* = 40)
	Mean (*SD*)/*n*%	Mean (*SD*)/*n*%
Mother's age	42.22 (4.96)	40.03 (4.17)
Parents age	43.15 (4.81)	42.83 (4.22)
Civil status		
Married/Cohabit	36 (85.7%)	33 (82.5%)
Single/Divorced	6 (14.2%)	7 (17.5%)
Education		
Elementary school	24 (57.1%)	20 (50%)
High school	8 (19.0%)	11 (27.5%)
University education	10 (23.8%)	9 (22.5%)
Professional status		
Skilled jobs	14 (33.3%)	12 (30%)
Unskilled jobs	16 (38.1%)	13 (32.5%)
Unemployed	12 (28.5%)	15 (37.5%)
Children’ age	9.21 (1.53)	9.35 (1.73)
IQ	97.53 (8.05)	100.04 (10.37)
Sex (*n*% boys)	38 (90.4%)	35 (87.5%)
Medication (*n*% yes)	31 (73.8%)	22 (55%)
ADHD children	22 (52.3%)	20 (50%)
ASD children	20 (47.6%)	20 (50%)

ADHD, attention‐deficit hyperactivity disorder; ASD, autism spectrum disorder; *SD*, standard deviation.

The primary aim was to evaluate the efficacy of an online‐based psychosocial program in improving parental domains (primary outcomes): specifically, stress levels, coping skills, and social support among parents of children with neurodevelopmental disorders (ASD and ADHD).

Regarding parent‐related primary outcomes, ANOVA results revealed statistically significant between‐group differences at post‐intervention, with the intervention group reporting statistically significant lower levels of total stress (*F*_1,80_ = 33.75; *p* < .01; $\eta_{p}^{2}$ = .29), and higher levels of positive coping (*F*_1,80_ = 25.66; *p* < .01; $\eta_{p}^{2}$ = .24) and perceived social support (*F*_1,80_ = 9.63; *p* = .025; $\eta_{p}^{2}$ = .10) compared to the control group. At follow‐up, these differences were maintained, with the intervention group showing statistically significantly lower stress levels (*F*_1,80_ = 32.58; *p* < .01; $\eta_{p}^{2}$ = .29) and statistically significantly greater positive coping (*F*_1,80_ = 30.02; *p* < .01; $\eta_{p}^{2}$ = .27) and social support (*F*_1,80_ = 7.06; *p* = .03; $\eta_{p}^{2}$ = .08) than the control group (see Table 2).

**Table 2:** Mix design ANOVA for outcome measures

Variables	Intervention group (*n* = 42)						Control group (*n* = 40)						Group statistics			
	Pre‐intervention		Post‐intervention		Follow up		Pre‐intervention		Post‐intervention		Follow up		Pre‐post		Pre‐follow	
	*M*	*SD*	*M*	*SD*	*M*	*SD*	*M*	*SD*	*M*	*SD*	*M*	*SD*	*F*_1,80_	$\eta_{p}^{2}$	*F*_1,80_	$\eta_{p}^{2}$
Parents																
T Stress	119.95	18.82	94.19	14.07	93.95	14.87	119.03	20.88	118.13	18.13	117.25	17.85	33.75**	.29	32.58**	.29
P Coping	8.67	3.45	13.76	3.90	13.69	3.49	9.85	3.07	10.43	3.05	10.57	3.00	25.66**	.24	30.02**	.27
S Support	28.93	11.73	39.62	10.15	38.14	10.63	29.03	11.88	28.13	10.52	28.02	10.51	9.63*	.10	7.06*	.08
Children																
SDQ‐Tot	26.17	5.69	20.67	6.64	20.62	6.38	25.30	5.88	24.95	5.84	24.97	5.81	10.38*	.11	10.96*	.12
BRIEF‐T	76.88	6.40	58.62	10.09	60.07	8.70	75.93	9.32	76.23	9.46	74.95	8.44	43.53**	.35	39.45**	.33
Sleep p	58.02	18.07	58.60	18.30	58.73	18.19	58.13	18.36	54.45	17.56	56.65	17.75	1.02	.01	1.11	.01
Wifirs‐F	14.69	6.29	9.43	3.99	10.00	3.96	13.93	5.89	13.70	6.09	13.85	5.56	11.47*	.12	10.65*	.11
LBS	24.60	8.26	25.48	7.28	25.26	6.80	25.35	7.72	25.33	7.85	25.22	7.62	0.17	.00	.13	.00

BRIEF‐T, Global Executive Composite Index; LBS, Learning Behavior Scale; P Coping, positive coping; S Support, total social support; SDQ, Total Strengths and Difficulties Questionnaire; Sleep p, total sleep problems; T Stress, total stress; Wifirs‐F, Wifirs Family Subscale.

*p < .05; **p < .01.

Although within‐group changes were not the primary focus, effect sizes for the intervention group indicated substantial improvements in total stress (*d* = 1.49 at post‐intervention; *d* = 1.50 at follow‐up), positive coping (*d* = −1.52 at post‐intervention; *d* = −1.49 at follow‐up), and social support (*d* = −0.97 at post‐intervention; *d* = −0.84 at follow‐up) (see Table 3). After applying the Holm–Bonferroni adjustment, all reported *p*‐values remained statistically significant, supporting the robustness of the findings.

**Table 3:** Effect sizes of change for outcome measures

	Intervention group (*n* = 42)			Control group (*n* = 40)				
	Pre‐post	Pre‐follow		Pre‐post		Pre‐follow		
Parents	Mean dif. Cohen *d* [95% CI]							
T Stress	1.49	[0.85, 2.12]	1.50	[0.87, 2.13]	0.05	[−0.48, 0.58]	0.10	[−0.42, 0.63]
P Coping	−1.52	[−2.19, −0.85]	−1.49	[−2.11, −0.88]	−0.17	[−0.75, 0.40]	−0.21	[−0.72, 0.29]
S Support	−0.97	[−1.73, −0.22]	−0.84	[−1.61, −0.07]	0.08	[−0.65, 0.82]	0.09	[−0.67, 0.85]
Children								
SDQ‐Tot	0.90	[0.31, 1.50]	0.91	[0.32, 1.51]	0.06	[−0.51, 0.63]	0.05	[−0.51, 0.61]
BRIEF‐T	2.07	[1.23, 2.91]	1.90	[1.15, 2.66]	0.03	[−0.72, 0.65]	0.11	[−0.51, 0.73]
Sleep p	−0.03	[−0.52, 0.46]	−0.04	[−0.53, 0.44]	0.20	[−0.30, 0.71]	0.20	[−0.29, 0.70]
Wifirs‐F	0.97	[0.34, 1.61]	0.80	[0.27, 1.46]	0.04	[−0.55, 0.64]	0.02	[−0.55, 0.58]
LBS	−0.11	[−0.73, 0.49]	−0.08	[−0.67, 0.50]	0.03	[−0.62, 0.60]	0.02	[−0.58, 0.62]

The secondary aim was to examine changes in child‐related domains (secondary outcomes), such as social challenges, executive functioning, sleep quality, family relationships, and learning behaviors.

Statistically significant between‐group differences were observed at post‐intervention for several child‐related secondary outcomes (see Table 2). Specifically, children in the intervention group had statistically significant lower scores on the SDQ total difficulties (*F*_1,80_ = 10.38; *p* = .025; $\eta_{p}^{2}$ = .11), BRIEF executive function (*F*_1,80_ = 43.53; *p* < .01; $\eta_{p}^{2}$ = .35), and WFIRS family impact scales (*F*_1,80_ = 11.47; *p* = .021; $\eta_{p}^{2}$ = .12) compared to the control group. These between‐group differences remained statistically significant at follow‐up: the intervention group showed fewer social‐behavioral difficulties (*F*_1,80_ = 10.96; *p* = .021; $\eta_{p}^{2}$ = .12), reduced impairments in executive functioning (*F*_1,80_ = 39.45; *p* < .01; $\eta_{p}^{2}$ = .33), and statistically significant lower levels of family‐related difficulties (*F*_1,80_ = 10.65; *p* = .025; $\eta_{p}^{2}$ = .11) relative to the control group. These findings suggest that the INPSYD digital‐based intervention was associated with greater improvements in these child outcomes over time compared to the control group.

Although within‐group effect sizes in the intervention group were large for SDQ total difficulties (*d* = 0.90 at post‐intervention, *d* = 0.91 at follow‐up), BRIEF executive functions (*d* = 2.00, *d* = 1.90), and WFIRS family impact (*d* = 0.97, *d* = 0.80), the primary focus remains on these observed between‐group effects (see Table 3). No statistically significant between‐group differences were found for sleep problems or learning behavior (LBS) at either post‐intervention or follow‐up, and the associated effect sizes were small.

## Discussion

The aim of this study was to analyze the efficacy of the INPSYD program in families of children with neurodevelopmental disorders (ASD and ADHD). Overall, the intervention program had a positive impact on most of the areas considered in this study.

Regarding parent outcomes, statistically significant improvements were observed in the intervention group compared to the control group. Parents in the intervention group reported a substantial reduction in parenting stress and notable increases in the use of positive coping strategies and perceived social support at post‐intervention and follow‐up. These improvements demonstrated large effect sizes, particularly for parenting stress and positive coping. These results align with findings from other studies reporting statistically significant benefits of psychosocial interventions. For instance, Yang et al. (2024) found that such interventions effectively help mothers reduce emotional burnout, lower the risk of depression, increase stress awareness, and enhance coping strategies and resources, particularly for chronic conditions like ADHD and autism. Similarly, Marquet‐Doleac et al. (2024) highlighted the positive effects of behavioral parent training on parental stress, feelings of efficacy, and reductions in negative educational behaviors. However, their findings regarding overall parental stress revealed mixed outcomes, suggesting variability across studies.

Other research underscores the value of combining behavioral training with problem‐solving strategies to alleviate parenting stress. Merriman, Burke, and O'Reilly (2020) emphasized that providing parents with accurate, factual information about ASD statistically significantly reduced stress and anxiety. Parents often reported that gaining a better understanding of their child's behavior through psychoeducation provided relief and a sense of empowerment.

Conversely, some studies report inconsistent results regarding the impact of psychoeducation alone. For example, Dahl et al. (2020) noted that none of the reviewed studies demonstrated significant effects of psychoeducation on parenting stress. They argued that psychoeducation often delivers diagnosis‐specific information, such as details about ADHD, but fails to address broader issues, such as daily functioning or strategies for managing the general stress of parenting a child with attention and behavioral challenges. This narrow focus may account for the inconsistent outcomes associated with psychoeducational interventions. These findings emphasize the necessity of integrating psychoeducation with broader support mechanisms, such as stress management training and problem‐solving strategies, to comprehensively address the complex challenges faced by parents of children with neurodevelopmental disorders.

Statistically significant improvements were observed in children's behavioral and executive functioning, as measured by the Strengths and Difficulties Questionnaire (SDQ), Behavior Rating Inventory of Executive Function (BRIEF), and WFIRS family score. The intervention group showed reduced social‐behavioral problems, enhanced executive functioning, and fewer family impairments compared to the control group at follow‐up. These findings underscore the INPSYD program's effectiveness in addressing child‐related outcomes that directly influence family dynamics. However, no statistically significant changes were observed in children's sleep or learning behavior, suggesting a more targeted impact on specific developmental domains.

These results align with prior studies highlighting the effectiveness of psychosocial interventions in reducing behavioral problems and improving adaptive skills in children with neurodevelopmental disorders (Chacko et al., 2024; Yang et al., 2024). Improvements in executive functioning, a key challenge for children with ADHD and autistic children, are particularly critical, as they impact academic performance, emotional regulation, and family relationships (Chesterfield, Porzig‐Drummond, Stevenson, & Stevenson, 2021). The program's outcomes align with similar interventions targeting cognitive and self‐regulatory skills (Evans et al., 2018).

Contrarily, the lack of statistically significant improvements in sleep and learning behavior diverges from studies like Corkum et al. (2016), which reported statistically significant sleep improvements through behavioral interventions and psychoeducation. The absence of sleep‐focused content in INPSYD likely explains these results. Similarly, modest effects on academic and learning outcomes reported in other studies (DuPaul et al., 2019) reflect the need for individualized and specialized learning interventions that were beyond the scope of this program.

Technology offers a promising mechanism for improving access to evidence‐based treatments. Digital and synchronous interventions, particularly those implemented during and after the COVID‐19 pandemic, have demonstrated effectiveness in overcoming logistical barriers to care while enabling personalized treatment delivery (Tan‐MacNeill et al., 2021). Such approaches could enhance both efficiency and accessibility for families of children with neurodevelopmental disorders.

Given the chronic nature of ASD and ADHD symptoms, treatment strategies must address not only the child but also the broader family, school, and community contexts. Psychosocial programs incorporating psychoeducation, self‐regulation strategies, cognitive‐behavioral techniques, and social communication skills are essential for promoting socio‐adaptive behaviors (Ferrin et al., 2014; Gosling et al., 2022). Moving forward, it is crucial to develop evidence‐based methods that optimize treatment access and fidelity while evaluating the cost‐effectiveness of psychosocial interventions (Dahl et al., 2020; Sampaio, Nystrand, Feldman, & Mihalopoulos, 2024).

### Limitations and further research

This study has several limitations that should be addressed in future research. The small, homogenous sample, primarily drawn from similar socioeconomic backgrounds, limits the generalizability of findings. Including larger, more culturally and socioeconomically diverse samples could provide a more comprehensive understanding of the program's effectiveness. A key limitation of this study is the relatively small sample size, which may reduce statistical power and increase the risk of Type II errors. Although our sample size is comparable to other studies (Pan et al., 2023), it nonetheless limits the generalizability of the findings. Future studies should aim to replicate these findings in larger samples.

The reliance on parent‐reported measures introduces potential bias or social desirability effects. Incorporating objective assessments, such as teacher reports, observational data, or physiological markers, would enhance the validity of the results. Additionally, the follow‐up offered valuable insights into short‐term sustainability but did not assess long‐term outcomes. Extending follow‐up periods could better evaluate the intervention's durability and the need for booster sessions or additional support. A further limitation of the present study is that it did not evaluate the individual effects of each intervention component – namely, psychoeducation and stress management, cognitive‐behavioral techniques, and social and communication skills training. Although examining each component separately, as well as in combination, would offer valuable insight into their specific contributions, this was not the focus of the current research. Future research could adopt designs such as multiple baseline approaches to disentangle the effects of each module and better understand their individual and combined impact.

Lastly, as a digital intervention, the program assumes access to stable internet and technology, which may not be accessible to all families, especially those in underserved areas. Moreover, no formal feasibility study was conducted prior to the trial, underscoring the need for future research to assess the implementation of such interventions in populations with limited technological resources. Also, future research should consider collecting qualitative data on participants' engagement and satisfaction with the intervention. Although not within the scope of the present study, such information would offer valuable insights into implementation and user experience.

## Conclusion

The findings from this study demonstrate the efficacy of the online‐based INPSYD program in improving outcomes for both parents and children in families affected by neurodevelopmental disorders. Despite some limitations, the large effect sizes and sustained benefits observed at follow‐up highlight the potential of such programs to fill critical gaps in the provision of psychosocial interventions. Future research should aim to build on these findings by addressing the identified limitations and expanding the scope of the program to target a broader range of outcomes. By doing so, digital interventions like INPSYD could play a pivotal role in enhancing the well‐being of families facing the challenges of neurodevelopmental disorders.

## Funding information

This study was supported by the Spanish project PID2021‐128044NA‐100 (Ministerio de Ciencia e Innovación MCIN/AEI/10.13039/501100011033/FEDER).

## Conflict of interest statement

The authors have declared that they have no competing or potential conflicts of interest.

## Ethical considerations

The informed consent was appropriately obtained from parents. The present study was conducted in accordance with the guidelines of the Ethics Committee of the Universitat de València, with approval obtained in 2021 (UV‐INV_ETICA‐1905517).

## Data Availability

The dataset and SPSS analysis syntax have been uploaded to a permanent Zenodo repository Link (https://zenodo.org/records/15465144) and will be made publicly available upon acceptance of the manuscript.
